# It’s Premature to Encourage Working Cats for Rodent Control on Australian Dairy Farms

**DOI:** 10.3390/ani16030417

**Published:** 2026-01-29

**Authors:** Michael C. Calver, Heather M. Crawford, Tim Kurz, Jo Watson, Bruce L. Webber

**Affiliations:** 1 Environmental and Conservation Sciences, Murdoch University, Murdoch, WA 6150, Australia; m.calver@murdoch.edu.au (M.C.C.); heather.crawford@murdoch.edu.au (H.M.C.); 2 School of Psychological Science, The University of Western Australia, Crawley, WA 6009, Australia; tim.kurz@uwa.edu.au (T.K.); jo.watson@research.uwa.edu.au (J.W.); 3 School of Biological Sciences, The University of Western Australia, Crawley, WA 6009, Australia

**Keywords:** animal welfare, evidence-based decisions, farm cats, human health impacts, integrated pest management, one welfare, roaming cats, rodent control, stray cats, wildlife predation

## Abstract

Rodents are significant agricultural pests in Australia and internationally. Recent proposals to address the Australian problems by allowing farmers to obtain desexed cats, at tax-deductible cost, to control rodents on their properties are, superficially, attractive, especially given enthusiastic endorsement from 15 dairy farmers from nine properties in Queensland and New South Wales. We argue that far stronger evidence is required to take this solution seriously, and for broader ethical and regulatory reasons, it may not be appropriate even if evidence was forthcoming. Evidence via direct population monitoring or indirect monitoring using bait removal is first needed to show that rodents are effectively suppressed by cats and how many cats are required. Further supportive evidence could come from monitoring the diets of cats using techniques such as analysis of stomach contents or scats, collar-mounted video cameras, or stable isotope analysis of cat tissues such as whiskers. These techniques would also quantify any predation by farm cats on wildlife. Population monitoring of cats would confirm whether there is unwanted immigration of cats into the farm cat population, while economic modeling could compare the costs of a farm cat program to other rodent control options. In the absence of such data, promotion of cats as a solution to rodent issues on Australian farms remains unsubstantiated and is premature.

## Simple Summary

Recent claims that farm cats in Australia can successfully control rodents on dairy farms are intriguing, although they lack important data for a convincing case. In particular, successful rodent control is claimed without monitoring rodent numbers on dairy farms to confirm their reduction in the presence of cats. There were also no dietary data to confirm that desexed and properly provisioned cats did not threaten wildlife, and no original data to confirm changes in the home ranges of desexed cats. Monitoring of cat numbers did not continue for several years to see if cat numbers re-establish after they are set at a level approved by the farmer, and there was no economic modeling of the relative costs of cats versus a range of alternative control methods. While there is no doubt that some farmers think that having cats roaming on their farms brings benefits for rodent control, given the lack of data the claims are, at present, unjustified. As such, there is no case to be calling for tax incentives to support cats as working animals on farms.

## 1. Introduction

In two recent papers in Animals, based on interviews with 15 farmers from nine dairies in the Australian states of Queensland and New South Wales, Crawford and colleagues [1,2] argued that Australian dairy farmers valued cats on their farms for rodent control, believing that cats were more successful, cheaper, and less hazardous to wildlife than rodenticides. They claimed dairy farmers also valued the cats for companionship. Crawford et al. [1,2] concluded that cats should be recognized as working animals for purposes of tax deductibility and programs should be instigated to desex farm cats and to desex unowned cats in shelters and make them available to farmers for rodent control.

While the claims are intriguing, we find that Crawford et al. [1,2]’s enthusiastic endorsement of farm cats for rodent control in Australia is, at present, unsupported by the available data, and thus premature. They present clear data of the perceived value of cats to the 15 dairy farmers they interviewed; however, one might question the appropriateness of attributing the views of these farmers to the wider Australian dairy community, or the Australian farming community more broadly. Moreover, we have concerns that their methodology ignores important recommendations of thematic analysis, the approach they have used to identify and report on themes within their qualitative dataset. Additionally, nothing beyond anecdotal data is provided to show that cats reduce rodent numbers, can be dissuaded from attacking wildlife, remain close to farm buildings after desexing, or that after a desexing program cat numbers will not recover with immigration from surrounding areas. Furthermore, we contend that Crawford et al. [1,2]’s own data show that there are uncertainties about exactly how many cats are required per property and the relative costs of cats compared to other rodent control options. We also raise substantial concerns regarding the welfare of farm cats in Australia on the basis of extensive euthanasia reported in these data. Indeed, extension of the desexing programs they describe on a wider scale could involve the euthanasia of up to a quarter of the existing farm cat population, as well as potential movement of unsocialized shelter cats across the country with no clear provision for responsible ownership. A more detailed exposition of the main areas of concern with these two papers follows.

## 2. Cats and Rodent Control

### 2.1. Is There Strong Evidence That Cats Are Effective Rodent Controllers?

It is important to support the case for farm cats as tax-deductible working animals, either on their own or in conjunction with other options, as part of integrated pest management (IPM). The analyses of the interviews with 15 dairy farmers reported by Crawford et al. [1,2] show clearly that these farmers believed that the cats on their farms kept rodents under control. We argue that those opinions need support from empirical evidence of declines in rodent numbers, distribution, or activity in the presence of cats, which is not provided by Crawford et al. [1,2]. As we explain below, the empirical evidence available from other studies is far from conclusive.

One of the earliest studies of potential control of rodents by farm cats was conducted in 1953 by Elton, who observed that ‘although cats are very often kept on farms and other premises, there is much difference of opinion about their efficiency in keeping down rat infestations. Many farms have been seen where cats live in company with permanent rat populations, often of some size: and others where it is claimed that cats are responsible for rat scarcity’ ([3], p. 153). His assessment was that a high density of cats could be effective in keeping areas around farm buildings rat-free if the cats were introduced after complete extermination of rats by other means and fed only milk to encourage them to hunt. Elton further opined: ‘The quantity of cats if (sic) probably more important than their quality’ ([3], p. 155), despite developmental studies demonstrating that cats have to refine and specialize their hunting skills to tackle intelligent rats [4,5]. He noted, though, that although rat numbers were controlled around buildings, they were still present elsewhere on the farms. Elton’s points are endorsed by Bai ([6], p. 191), who concluded that: ‘… cats (if kept unfed) can keep the immediate environment rodent free, if the population is low and are successful in modifying the composition, but not the size of population (sic).’ Newsome [7] refers to Elton [3] as an illustration of a basic ecological principle that predators may control prey numbers when the prey population is already much reduced by another agency, but are unlikely to reduce a major infestation significantly.

More recent data on the efficacy of cats as rodent controllers are ambivalent. For example, as the practice of maintaining colonies of desexed cats has grown in response to the prevalence of unowned cats around human habitation, several authors have reported that rodents are actually more prevalent around cat feeding stations than elsewhere, with the rodents tolerating increased predation risk to access uneaten cat food [8,9,10,11,12]. Others express doubt on the efficacy of cats as rodent controllers, including questioning the value of anecdotal evidence of control success [13] or present data indicating that cats have limited success in rodent control [14]. Rodent behavior may be an important factor, with some authors finding that roof rat (also known as black rat) *Rattus rattus* activity can overlap highly with carnivores such as dogs and cats [15] and that *R. rattus* does not avoid cat and dog odors [16], whereas brown rats *Rattus norvegicus* are more likely to perceive cursorial carnivores as threats [15].

In contrast, Wijburg et al. [17] report that in urban areas of the Netherlands the presence of cats was a negative predictor of rodent occurrence. In a study of German livestock farms, Esther et al. [18] noted that dogs and cats as predators (they did not distinguish between the two) made a substantial difference to reducing rodent activity by over 30%, as measured by inactive bait boxes (i.e., no feeding by rats), in the absence of other rodent management. On farms in Swaziland (now known as Eswatini), dogs and cats together were successful in reducing rodent activities in homesteads, but neither predator alone affected rodent abundance [19].

Finally, some authors question whether any effect of cats on rodent numbers or activity arises from repellent odors rather than predation. If true, live cats would be unnecessary for deterring rodents from particular areas of a farm, with cat odors from fur, urine, or feces effective (see Hillar et al. [20] for an assessment of the repellent idea, and Stryjek et al. [21] for a more skeptical conclusion).

### 2.2. What Potential Problems Arise from Deploying Cats for Rodent Control?

Encouraging cats on farms to control rodents may cause problems with predation on wildlife, sanitation, and disease, as well as the welfare of the cats themselves. If cats attack rodents, they will likely attack wildlife too (e.g., [22]), although the farmers interviewed by Crawford et al. claimed that this was not substantial after the cats were desexed. Many of the claims made by Crawford et al. [1] regarding the effectiveness of their program in reducing predation on wildlife by farm cats seem counter to the cats’ potential effectiveness as rodent controllers. For example, if desexing programs on farms mean fewer kittens, according to Crawford et al. [1] this leads to resident cats being older, less likely to hunt, and more likely to find sufficient food in what the farmer provides. If so, will they not hunt and kill fewer rodents? Furthermore, if increasing supplemental food reduces hunting, will this not reduce predation on rodents? There is evidence that poorly fed dogs and cats may hunt more [23], although many well-fed cats still hunt [24], so Crawford et al.’s [1] logic for dismissing impact on wildlife by dairy cats is questionable.

With regard to cats’ toileting sanitation, Legge et al. [25] estimated that cat-dependent diseases associated with the pathogens *Toxoplasma gondii*, *Sarcocystis gigantea*, *S. medusiformis*, and *Bartonella henselae* cost Australia AUD 6 billion per year through costs to human health and livestock production. Minimizing cat density on farms, especially areas used for livestock production, was proposed as one significant mitigation, which is counter to encouraging cats for rodent control. Emerging diseases may also be a problem, with cats on farms potentially contracting avian influenza when fed unpasteurized milk from infected dairy cows [26] and, in turn, potentially transmitting it to humans [27].

Finally, it is important to consider the welfare of the cats. Given the advice to feed farm cats only milk [3] or not to feed them at all [6] to encourage the most effective hunting, would using cats for rodent control under such conditions be best for their welfare? Feeding farm cats at least something was common in surveys of farmers in Austria [28] and the UK [29], although some UK farmers distinguished between their own pet cats or farm cats on one hand and perceived feral (seen on the farm but not fed and not considered part of the farm) cats on the other [29]. Even if ‘working cat programs’ desex cats and provide occasional parasite prevention, working cats’ health is unlikely to be monitored as closely as that of pet cats, so their welfare is unlikely to be optimal [28,29].

### 2.3. Could Other Predators Provide Effective Control with Fewer Problems?

If predation is effective, either solely or in combination with other techniques, need it be provided by cats? As noted by Esther et al. [18], dogs are also an option (especially for rats), while Mahlaba et al. [19] found a combination of dogs and cats effective in rodent control. Of course, farm dogs should be contained when not working to avoid interactions with wildlife. Other authors note a range of potential predators [6], including encouraging owls in agricultural settings for rodent control [30,31,32]. Of these, we support work assessing the value of owls, using translocated or captive-bred owls that, with appropriate nest box provision, could establish and breed, with potential for significant rodent control [31,32,33,34] without the disease and welfare problems associated with cats or rodenticides. Even measures as simple as providing perch sites can attract owls and other predatory birds [35]. We acknowledge, though, that just as cats attack wildlife, so predatory birds may hunt species of conservation concern [36].

### 2.4. The Place of Predators in Integrated Pest Management (IPM)

IPM, including combinations of two or more of repellents, trapping, rodenticides, hygienic management, habitat modification, and predators, is proposed or has been found to be successful in several contexts [37,38,39,40]. Ideally, IPM achieves good control while minimizing side effects, with farmers’ attitudes and good farm advisory services important factors in success [41]. For example, Esther et al. [18] found that following implementation of an IPM program, the added effect of dogs and cats was just over 5%, which is a small benefit of these predators that we consider is outweighed by the problems they may cause. Accepting that reduced rodenticide use is an important goal to avoid problems with resistance and the risk of secondary poisoning [42], it is also important to provide sound data on the efficacy of IPM to discourage excessive use of rodenticides when less hazardous options achieve good results [43]. The New South Wales Department of Primary Industries provides a detailed code of practice, together with standard operating procedures, while there is also a national industry code of best practice for rodent management by pest controllers [44,45]. We were surprised that these documents were not cited in either of Crawford et al. [1,2]

### 2.5. How Good Is the Evidence Provided by Crawford et al. for Rodent Control by Cats?

Overall, the evidence given in Crawford et al. [1,2] for cats controlling rodents on the study farms is anecdotal, with no quantitative evidence of the level of rodent activity. The anecdotal reports may be accurate, but given the varying assessments in the literature on the efficacy of predators in controlling rodents, quantitative data are needed before cats on farms for rodent control in Australia can be considered as a management strategy. We also note that claims of effective rodent control appear to have been made over a wide range of cats/property after the desexing program (3–79, mean 23.0, see Crawford et al. [1], Appendix A, Table A1). Assuming the claims are accurate, it appears that few cats are actually needed for farmers to perceive a benefit, although this observation may be affected by farm size. For comparison, in 2022 the mean number of cats on a sample of full-scale Danish farms (i.e., where the farmer earns a living from the property) was 1.96 (range 0–11), while the number of cats on a sample of Danish hobby farms was 1.42 (range 0–10) [46]. On Austrian farms, the mean number of cats per farm was 4.6 (range 1–22) [28], while in a UK study the mean was 0.65 (range 1–27) for farm cats, defined as gaining most of their food from hunting and living mainly outdoors or in outbuildings, although there could also be pet cats (mean 0.49, range 1–8, living mainly indoors and fed by a family member) or ‘feral’ cats (mean 0.15, range 1–27, living around the farm, but not considered part of the farm) [29]. The number of cats on the dairy farms studied by Crawford et al. [1,2] appears large by international standards.

## 3. Modeling the Relative Costs of Management Options

The case for tax deductibility of the costs of maintaining farm cats in Crawford et al. [2] is not supported in the paper by quantitative analysis of all costs and benefits. For example, the benefits arising from specific numbers of cats on a farm (perhaps related to farm area) in terms of reductions in production losses, equipment damage and so on, as well as the costs of maintaining the cats, need to be parameterized for entry into cost–benefit models so that the economic benefit of the cats can be assessed. We have not undertaken such work, nor do we claim that the results would oppose keeping farm cats for rodent control. We do, though, claim that it is reasonable to expect such data from a representative number of farms in making a case for tax deductibility for working cats. We were disappointed to find the data missing from Crawford et al. [1,2], especially at a time when there are calls in the academic taxation literature for a re-examination of the taxation regulations around livestock in Australia more broadly [47,48]. Excellent models for how to undertake such a study are provided in work on *T. gondii* control in piggeries (e.g., [49,50]).

## 4. Cat Ecology and Behavior

Crawford et al. [1,2] make large, essentially unsubstantiated, claims regarding the effects of the desexing program on cat ecology and behavior, specifically stabilizing or reducing populations, decreased hunting behavior lowering impacts on wildlife, and reduced roaming. We consider each claim in turn.

### 4.1. Cat Population Reduction or Stabilization

Appendix A, Table A1 of Crawford et al. [1] indicates that rehoming of cats plus euthanasia of seriously ill or unwanted cats reduced the number of cats on the nine properties by between 0 and 61.3%, with a mean reduction/property of 32.8%. Putting aside the point that this mean reduction provides little insight of value given such high variance, without medium to long-term monitoring of cat numbers, it is unclear whether these reductions will be maintained, although work in the USA supports the concept that farms where cats are desexed have lower cat populations [51]. We assume that, in general, farms are not closed populations of cats, so new cats may immigrate following control [52]. Such immigration can frustrate attempts at long-term population stabilization, a problem noted with some Trap–Neuter–Return program (TNR) studies in urban areas [53]. Before desexing could even be considered as an effective strategy for cat population control at a landscape scale, co-operative cat management across a range of contiguous farms or other landholdings, or cat control on adjacent conservation reserves or crown land, would be needed. Such coordination is likely to prove challenging. Furthermore, monitoring for a decade or more is needed before concluding that the program maintains cat numbers at the level desired by the farmers, as well as the level of repeated trapping and desexing required. This agrees with the call by Ramírez Riveros [54] for detailed studies to evaluate control effectiveness in a range of settings. In Crawford et al. [1,2], the number of cats remaining on each dairy farm after desexing was determined by the surveyed farmers, not through any empirical assessments.

### 4.2. Decreased Hunting Behaviour and Lowering Impacts on Wildlife

Crawford et al. [1] (p. 3) claim that: ‘These behavioral changes indicate that sterilization not only reduces cat populations but improves cat welfare and helps to decrease their potential impact on wildlife,’ citing five studies in support. Concentrating on the claim ‘to decrease potential impact on wildlife,’ none of the five references includes any data on wildlife impacts. We searched each reference for the words ‘wildlife’ or ‘predation,’ finding:

- Crawford et al. [55]—No data on hunting of wildlife were presented, but the opinion that desexed cats are less of a hazard to wildlife was reiterated.
- Nutter [56]—No data on hunting of wildlife were presented, although the possibility was mentioned several times.
- Gunther et al. [57]—Not only are there no data on hunting and wildlife, but the words wildlife and predation do not appear in the paper at all.
- Spehar and Wolf [58]—No relevant data are presented; the word wildlife appears once in the context that some consider free-roaming cats an issue for wildlife. Predation does not appear.
- Centonze and Levy [59]—Wildlife is mentioned once, in the context that no TNR colonies occurred ‘… on parks or wildlife preservation areas’ (p. 1631). The fact that care was taken in placing colonies implies a recognition that such colonies may threaten wildlife. The term ‘predation’ does not appear.
- Scott et al. [60]—Not only are there no data on hunting and wildlife, but the words wildlife and predation do not appear in the paper at all.

The link between cat desexing and reduced impacts on wildlife is thus not substantiated by Crawford et al. [1] beyond the subjective opinions of farmers, nor do their primary references give support. Crawford et al. [2] (p. 3) make a similarly strong claim: ‘Providing care for cats, such as sterilization and food, reduces their need to predate to meet their energy needs and reduces the population size over time, and hence reduces impacts on native wildlife’, citing two studies in support:

- Silva-Rodríguez and Sieving [23]—Unlike all the other studies cited in support of the hypothesis that well-fed cats hunt less, this paper includes data showing statistically significant declines in predation of vertebrates by cats fed adequately compared to those that are not. Nevertheless, some hunting was still reported for well-fed cats.
- Ferreira et al. [61]—The word wildlife appears twice in this paper and predation once; the authors present no data indicating reduced predation by desexed or well-fed cats, although they do demonstrate declines in home range after desexing that might plausibly lead to reduced predation.

By contrast, studies of the predatory behaviour of owned cats, many of which are fed daily and desexed, confirm that they can be prodigious hunters [24,62,63,64]. In the specific case of free-roaming cats on livestock farms in the USA, Kitts-Morgan et al. [65] presented data that native wildlife were eaten. When discussing the attitudes of Austrian farmers to desexing of their cats, Heizmann et al. [28] cited studies stating that some farmers feared that cats’ hunting abilities would be reduced. In Japan, diet analysis revealed that unowned cats relied on human subsidies but also predated on wildlife in adjacent forested areas, with the subsidies increasing the likelihood of predation by cats [66]. In another telling example, Greenwell et al. [67] report how predation by a single, desexed, unowned cat was primarily responsible for the abandonment of a nesting colony of the threatened coastal seabird the Australian Fairy Tern *Sternula nereis nereis*, with 111 nests failing and six breeding terns killed. Although at least some data-based studies show that feeding cats reduces, but does not eliminate, predation [23,68], others found no effect of feeding on predation [69]. Cat age, nocturnal containment, and provision of toys may also reduce hunting [70], although these are all difficult to implement in the case of farm cats and may interfere with the primary aim of rodent control. Essentially, cats’ evolution as opportunistic hypercarnivores means that if they are allowed outside there is a risk to wildlife [69]. In view of the literature, studies of the diets of farm cats in Australia under different conditions are warranted before considering any endorsement of a working cat program.

Crawford et al. [1,2] present anecdotal reports from farmers who noticed less predation on wildlife by cats after the desexing program. The problem with these reports is that we know from studies where owner records of predation are compared with the irrefutable evidence provided by collar-mounted video cameras that owners detect at best only one-third of predatory episodes, that the prey they identify are not representative of the real kills or mutilations attributable to their cats, and that the prey seen are not representative of the true relative proportions of those attacked [71,72]. A similar standard of evidence is required to establish that desexed cats in a working cat program hunt less wildlife.

While work with collar-mounted cameras can be challenging, cat diets can also be assessed using techniques such as those described in [73]. Crawford et al. [22] present a specific example based on examination of the stomach contents of culled unowned urban cats, finding that, of the sample of 188, over half had eaten fauna and 57.5% had swallowed refuse that threatened intestinal damage or blockages. The option of examining stomach contents was available in the program reported by Crawford et al. [1,2], given the 100 cats euthanized. There may also have been an opportunity to examine any scats deposited in traps. Such data would be useful in obtaining a snap-shot of cat diet before desexing/culling on dairies and repeated assessment of scats would allow comparison of diets before and after intervention.

While predatory effects of cats on wildlife garner most attention, transmission of diseases, especially toxoplasmosis, is the subject of increasing study. Macropods are highly susceptible to toxoplasmosis [74]; there are also concerns about transmission to humans when game is hunted for meat [75,76]. While much work remains to be done, studies to date have identified toxoplasmosis as a significant cause of mortality in macropod populations [77].

### 4.3. Reduced Roaming

While it is plausible that desexed cats may roam less, most studies of home range in cats compare the home ranges of entire and desexed animals without reference to the habits of desexed animals before surgery. Ferreira et al. [61] implemented such a before/after study, concluding that desexed male cats had reduced home ranges of up to 80% as well as reductions of c. 25% in their overall activity. Such reductions may reduce predation on wildlife by farm cats, although Crawford et al. [1,2] overlooked a significant opportunity to collect relevant data in an Australian context by determining the home ranges of cats in their study before and after desexing, or to collect data on the diets of the cats as well under these conditions. This is important, given that in another Australian study the largest home ranges were reported for a desexed male cat and a desexed female cat [78].

## 5. Extrapolating from a Small-Scale Study

Crawford et al. [1,2] do describe the limitations of their study in terms of sample size, sample representativeness, and possible self-reporting bias, although we were surprised that no details were given on the demographics (e.g., age, gender) of the respondents. To this lack of detail we add the absence of quantitative data on rodent populations, the diet of the cats, and cat roaming behavior, as well as the lack of economic modeling of the costs of a range of control options, delivered either singly or in an integrated program. We are also concerned about inferences drawn from the particular social research methods used. To be clear, we do not wish to in any way undermine the utility or credibility of qualitative methods. Indeed, our concerns here are partly motivated by a desire to avoid seeing inappropriate conclusions being drawn from such methods precisely because such assertions risk undermining their (wider) credibility.

It is claimed, in Crawford et al. [2], that their analytic approach draws on the seminal thematic analysis writings of Braun and Clarke, citing their 2012 handbook chapter [79], although we would point the reader to many more developed and recent sources (e.g., [80,81]). In our reading of Crawford et al. [1,2], we are concerned by the extent to which some of the fundamental assumptions and recommendations of Braun and Clarke’s writings/approach to qualitative research seem to have been overlooked. One example is the absence of an appendix with an Interview Guide including specific questions to complement the topics presented in the Methods. A second example is the absence of a reflexivity statement by the authors reflecting on the background and assumptions they brought to the study that may have shaped the research. Such an appendix or statement would also perhaps have clarified how the goals of the charities funding the work aligned with the intent and development of survey questions for dairy farmers.

Most crucially, for our current argument, neither Crawford et al. paper offers a particularly clear articulation of the epistemological/ontological position of the research. The authors refer to having adopted ‘a phenomenological approach [that] allows researchers to understand a phenomenon from the perspective of the people involved’ and state that this ‘was an appropriate way to understand and explore the lived experience of dairy farmers who had cats on their farms and who had cats sterilized through one of two free sterilization programs’ ([1], p. 4). However, the (types of) conclusions being drawn across these papers do not appear to match well with this orientation.

By their own account, these interviews have captured the lived experience of a small group of dairy farmers who already have working cats around their farms and who have participated in a free sterilization program. Members of this group have presumably embraced cats (to some extent), potentially formed relationships with individual animals, and are actively seeking to manage cats’ presence around their farms. While it may well be of interest to understand the experience of those in this group, we do question the extent to which the authors choose to draw conclusions about (all) ‘Australian Dairy Farmers’ based on these interviews. While the authors acknowledge their small sample, we question the extent to which they fully acknowledge the *specificity* of their sample. We would also point out that there was a degree of awkward disjuncture at times between the purported phenomenological approach being adopted and the ontological status attributed to the observations made by the participants. In such an approach, it would have been more appropriate to interpret the utterances of the interviewees as insights into these farmers’ own lived experiences—experiences that could then have been contextualized critically by the authors with relevant literature. To our mind, the authors instead chose to reinforce these findings with somewhat filtered interpretations and an (arguably) biased selection of literature. In essence, our concern here relates to what Braun and Clarke [81] (p. 1) refer to as the danger of ‘swim[ming] unknowingly in the waters of positivism’. There is a risk that Crawford et al. [1,2] are making positivist claims using data that are not well suited to providing appropriate evidence for such claims. If one wishes to make claims regarding ‘what dairy farmers think’ then this really requires a representative quantitative survey of many dairy farmers. Such a survey would consider sample size, representativeness, conflicts of interest (e.g., if there were rewards or incentives for participation), reasons for inclusion or exclusion from surveys, a decision matrix for cat euthanasia, and careful definition of terms (e.g., is ‘rehomed’ equivalent to ‘adopted?’). Qualitative thematic analysis of 13 interviews is a perfectly acceptable (and indeed highly desirable) method for opening up new theoretical insights into the kinds of things that dairy farmers *might* (en masse) think about an issue. However, it does not allow one to confidently draw conclusions about the validity of those theories. Our concerns are not with the use of qualitative methods, but with the disconnect between the methodological approach and the nature of the conclusions drawn. Crawford et al. present findings from a small, specific group as broadly representative without sufficient critical analysis or epistemological clarity, resulting in an overreach in their conclusions.

## 6. Cat Welfare Concerns

Appendix A, Table A1 of Crawford et al. [1] documents that 16.3%, or approximately one in six, of the cats in the study were euthanized for undisclosed welfare reasons. We also note that Crawford et al. [2] refers to the same dataset, but with a discrepancy. Both papers refer to a mean of 44 cats with a range of 3–175 cats across the properties before the desexing program, but Crawford et al. [2] refers to a mean of 18 and a range of 3–60 post-desexing whereas the post-desexing data in Appendix A, Table A1 of Crawford et al. [1] have a mean of 23 and a range of 3–79. Whichever is correct, these figures are high for TNR studies. Luzardo et al. [82] (p. 9) claimed that ‘… euthanasia rates due to serious diseases are usually very low in community cats (less than 1%), and their body condition is generally good.’ Although that claim was not backed by sources, considering euthanasias and unplanned deaths together, Levy et al. [83] reported only 1% and Spehar and Wolf [58] only 0.5%. A higher euthanasia rate of 17.4% was reported for the Ocean Reef Community Association ORCAT TNR program in Florida, USA [84], likely because of the policy of euthanizing feline-retrovirus-positive cats. Eckman and Hiby [85] also claim welfare success in TNR, listing 10 examples in support.

Overall, we are concerned that the prevalence of euthanasia for health reasons in the Crawford et al. sample may indicate significant health and welfare concerns for cats on Australian dairy farms. Problems became more acute as the number of cats on a property rose, with a highly significant correlation between the initial number of cats on a property and the number euthanized for welfare reasons. While desexing may improve welfare outcomes, especially by reducing fighting with its potential for injury and disease transmission [86,87], other serious feline infectious diseases may be transmitted via direct contact or fecal contamination, which are unaffected by desexing [88]. In this context, there are documented cases of poor welfare outcomes for cats in TNR colonies (e.g., [89,90,91]) so any improved welfare may not be long-term. Studies on farms in Austria [28] and the UK [29] report vaccination rates of farm cats lower than 10%, although rates of worming and flea treatments were higher. In the Austrian study, many farmers were unwilling to seek veterinary intervention if their cats fell ill, which might also be a welfare concern relevant to farm cats in Australia. Based on the high euthanasia prevalence in Crawford et al. [1], which implies high disease or injury prevalence, vaccination rates may also be low in Australia. Crawford et al. [1,2] report that a further 9.2% of cats were euthanized because the farmer considered their numbers excessive. Not only did this lift the total euthanasia rate to 25.5%, or just over one in four cats, it is against the ethical philosophy of TNR to euthanize healthy cats [92]. Previous to Crawford et al. [1,2], five of the authors of those papers took a position opposing such euthanasias, calling them ‘trap–adopt–kill’ [55]. Furthermore, Crawford et al. ([1], p. 15) advise: ‘However, where there are existing healthy cats in excess of farmer needs, rather than ask farmers to kill cats before the trapping team arrives or veterinarians being required to kill healthy cats, these should be sterilized, returned to the farm, and then be advertised through farming channels and relocated over time to other farms requiring working cats that are sterilized.’ It is unclear why the authors did not follow their own recommendation and instead euthanized healthy farm cats.

Deploying the approach described in Crawford et al. [1,2] on a national basis would mean, based on the data they present, the euthanasia of approximately a quarter of the farm cat population. The ethical basis of this proposal should be considered, especially if any effect of cats on rodent control could be accomplished largely by cat odors as deterrents or IPM. It also questions the welfare basis for transferring cats from shelters to farms, where it may be difficult to sustain their welfare.

## 7. Applying One Welfare

One Welfare argues that the welfare of humans and non-human animals is intertwined and that maximizing both increases environmental sustainability [93]; it is an important part of the argument in Crawford et al. [1] but not Crawford et al. [2]. As stated, few would argue with the concept, although there are practical problems in implementation not considered in Crawford et al. [1] regarding the definition of welfare, the scoping of the people and animals potentially affected by actions, the definition of sustainability adopted, and how to resolve conflict if improving the welfare of one group reduces the welfare of another.

Considering animal welfare alone, there is no consistent definition [94]. Some definitions focus on animals’ physical and mental well-being (e.g., [95]), others address the challenging issue of the welfare of the individual versus that of a population [96], while there is a movement extending back to last century to include social and ethical implications beyond an animal’s immediate well-being [97,98]. Miribung et al. [99] consider that defining welfare requires a transdisciplinary approach, extending across legislation, natural science, and ethics, although this may challenge some preconceptions. Taking an extended definition that includes ethics, for example, would require a consideration of the ethics of desexing which, as Sandøe et al. [100] explain, is problematic under a deontological or animal rights argument. Thus, to argue from a One Welfare base, a clear definition of welfare is essential. Even then, it could be contested.

One Welfare also requires scoping the boundaries of what groups of people and what animals are to be included. Crawford et al. [1] are reasonable in including the cats on dairy farms, the dairy farmers, and the wildlife that might interact with the cats. One might also argue for including consumers of farm products who might have concerns regarding hygiene on the properties, wildlife advocates who may suffer distress over cats’ predatory activities, or even animal welfare advocates unsettled by the implications of the high levels of euthanasia implicit in the proposed program. Serious consideration should be given to the welfare of the cats themselves, especially given the potential conflict between provisioning on one hand and the likely effectiveness of cats as hunters on the other. A case could be made for considering the welfare of the rodents too, considering the likelihood of death versus repelling and the means of death when it occurs [101]. We recognize that including all these interest groups in a single study taking a One Welfare approach may be impractical. We do, though, argue that any case based on One Welfare should acknowledge the range of interests involved before giving a rationale for the groups retained in the final analysis. On this basis, we argue that the One Welfare applications in Crawford et al. [1] are not scoped adequately.

Furthermore, Crawford et al. [1] do not consider the complexities of assessing environmental sustainability, especially the tensions that can arise in balancing animal welfare needs in agriculture with larger sustainability goals. We align with the view that any case based on sustainability should be clear on what is being sustained, over what geographic area and what timescale, for whose benefit, at whose cost, and how sustainability will be measured. Wawrzyniak [102] explores the tensions between assessing sustainability and agriculture in general.

Lastly, we do not see how One Welfare resolves situations in which maximizing the welfare of one group compromises the welfare of another. Should the program as described proceed if, for example, it is shown that some wildlife deaths are inevitable and wildlife advocates are distressed? Should wildlife welfare have more or less influence than cat welfare or human welfare (a problem considered by Díaz et al. [103]), or even the welfare of the rodents themselves [101]? Should one consider the broader ethical view that the dairy industry itself is in need of significant animal welfare reform [104,105]? We do not claim to have answers for every point raised. We do, though, argue that any case purportedly based on One Welfare should define welfare, justify the groups included in the scope, define sustainability in the context of the proposed actions, and recognize potential conflicts with suggestions for resolution.

## 8. Regulatory Concerns

Returning desexed cats to the environment is illegal across Australia under diverse regulatory provisions covering biosecurity and animal abandonment [106]. Furthermore, many jurisdictions place conditions on cat ownership, requiring one or more of desexing, microchipping, registration with the local council, or wearing a collar with an ID tag. There are also limits placed on the number of cats owned by individuals, with council permission needed to own more than two, or sometimes three or four, cats. How would cat ownership by dairy farmers be regulated in Australia? If their cats are not pets, then are they subject to different rules, if any? How would any rules be enforced? While some dairy farmers may value the cats’ companionship, would they provide cats with sufficient food and timely health care? Or would dairy farmers be considered irresponsible owners under Australian law? Crawford et al. [1] suggest that cats kept on farms for rodent control should be exempt from registration and permit costs. What happens, then, if a cat is a poor hunter, or is valued largely for companionship?

There are many legislative acts at State and Commonwealth levels in Australia which encourage the lethal control of feral cats for protection of the environment, biodiversity, agriculture, and human health [107,108]. For example, in Western Australia, feral cats are a ‘declared pest species’, meaning that landholders pay a tax that contributes to lethal control of cats on Crown land and private properties. It is illegal to move or release declared pests, and their presence must be reported to authorities. Farmers working hard to remove cryptic feral cats from vast properties may not welcome neighbors harboring multiple cats that are able to roam freely for rodent control, while claiming a tax deduction. Any cats naturally recruited onto farms would possibly be feral and therefore subject to lethal control. Offering tax incentives for ‘working cats’ would inevitably cause confusion in Australian communities and, we expect, undo any gains in local feral population reduction.

Additionally, the suggestion by Crawford et al. [1] to place cats languishing in shelters onto dairy farms for rodent control seems like a thinly veiled attempt to reduce shelter numbers without euthanizing cats that may be inappropriate for rehoming. However, the desexing program described by Crawford et al. [1,2] euthanized a quarter of existing farm cats, so transportation of cats from shelters to farms, or from farms with too many cats to those without/too few, is merely deferring responsibility for euthanasia and the date at which it occurs. Surely as many cats as possible should be placed in homes where their welfare is a priority? All organizations working for the betterment of cat welfare inevitably encounter the issue of how best to manage the small proportion of cats that will never be in good health or have a temperament unsuitable for rehoming. We argue that endorsing the placement of such cats on dairy farms is not the solution.

## 9. Conclusions

While we remain open to being convinced on the merits of the proposals in Crawford et al. [1,2] for countries where TNR might be considered suitable, at present we question the strength of the case that has been made, particularly in the Australian context. The diverse concerns that we have raised regarding these two papers might be more than one research group could address in a single study. However, reporting of any part should acknowledge the limitations of a smaller study and indicate awareness of the larger picture and the other data needed for a convincing, comprehensive assessment of any impacts of cats on rodents.

We also note that as part of the peer review process (which is open for [2] and available to view online), many of the issues covered in this response were raised as points to address in earlier submissions of [2]. More detailed attention to those comments at the review stage, especially with editorial guidance and allocation of adequate time to address them, would have strengthened the paper. Neglect of these points may have negative reputational consequences [109,110,111,112]. In the spirit of such transparency, and to provide readers with a better understanding of the nuances of this exchange, we opted for an open review of our paper.

At a minimum, the case being made by Crawford et al. [1,2] would require data showing that the presence of cats reduces rodent numbers, that the effect of cats can be maintained while avoiding substantial predation on wildlife or disease risks, that cats’ home ranges are reduced by desexing, that cat populations do not rebound with immigration following a desexing program and that tax deductibility is supported by financial modeling. Moreover, Crawford et al. [1,2] do present data indicating poor welfare of farm cats, especially when there are many on a property. Data are needed to show that these outcomes improve following desexing programs, as well as indicating the minimum number of cats needed to achieve any benefits in rodent control. More quantitative, representative data on dairy farmers’ attitudes and perceptions are also required to understand the logistical and social psychological factors that may affect the practicality and (potential unintended) consequences of implementing Crawford et al.’s [1,2] recommendations. One Welfare, if used as part of the case, should be more rigorously defined and evaluated. Even if these data were available, for broader ethical and regulatory reasons it may never be justifiable to allow cats to roam on farms in an attempt to control rodents in Australia. What is clear is that any calls for investment in a ‘Barn Cat Program’ or tax deductions for working cats on Australian farms are premature and, importantly, unsubstantiated.

## Abbreviations

The following abbreviations are used in this manuscript:

IPM	Integrated Pest Management
